# Cubic and Hexagonal Mesophases for Protein Encapsulation: Structural Effects of Insulin Confinement

**DOI:** 10.1021/acs.langmuir.1c01587

**Published:** 2021-08-09

**Authors:** Paola Astolfi, Elisabetta Giorgini, Diego Romano Perinelli, Francesco Vita, Fabrizio Corrado Adamo, Serena Logrippo, Marco Parlapiano, Giulia Bonacucina, Stefania Pucciarelli, Oriano Francescangeli, Lisa Vaccari, Michela Pisani

**Affiliations:** † Dipartimento SIMAU, Università Politecnica delle Marche, via Brecce Bianche, 60131 Ancona, Italy; ‡ Dipartimento DISVA, Università Politecnica delle Marche, via Brecce Bianche, 60131 Ancona, Italy; § Scuola di Scienze del Farmaco e dei Prodotti della Salute, Università di Camerino, Via Gentile III da Varano, 62032 Camerino, Macerata, Italy; ∥ Scuola di Bioscienze e Medicina Veterinaria, Università di Camerino, Via Gentile III da Varano, 62032 Camerino, Macerata, Italy; ⊥ Elettra-Sincrotrone Trieste S.C.p.A., S.S. 14—km 163.5, 34149 Basovizza, Trieste, Italy

## Abstract

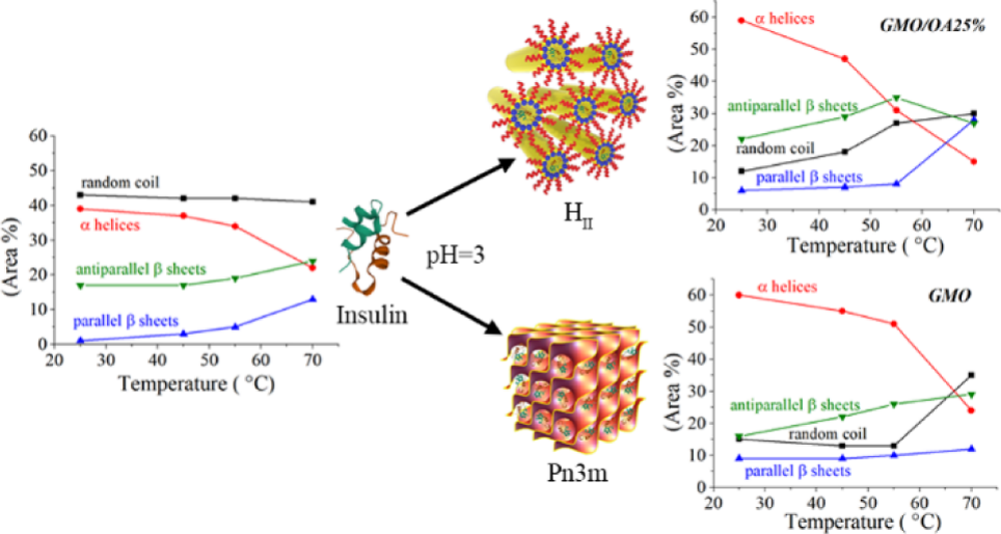

Monoolein-based cubic and hexagonal mesophases were investigated as matrices for insulin loading, at low pH, as a function of temperature and in the presence of increasing amounts of oleic acid, as a structural stabilizer for the hexagonal phase. Synchrotron small angle X-ray diffraction, rheological measurements, and attenuated total reflection-Fourier transform infrared spectroscopy were used to study the effects of insulin loading on the lipid mesophases and of the effect of protein confinement in the 2D- and 3D-lipid matrix water channels on its stability and unfolding behavior. We found that insulin encapsulation has only little effects both on the mesophase structures and on the viscoelastic properties of lipid systems, whereas protein confinement affects the response of the secondary structure of insulin to thermal changes in a different manner according to the specific mesophase: in the cubic structure, the unfolding toward an unordered structure is favored, while the prevalence of parallel β-sheets, and nuclei for fibril formation, is observed in hexagonal structures.

## Introduction

Lyotropic liquid crystalline (LLC) mesophases, particularly cubic and hexagonal ones, have been extensively studied as vehicles for molecules of different size and polarity such as small drugs, vitamins, peptides, proteins, and nucleic acids.^1−6^ In fact, encapsulation permits the control of the release of the enclosed molecule in space and time, thus possibly allowing targeted therapies, and represents an efficient strategy for biomolecule stabilization. Recently, we reported the use of phytantriol (PHYT) or monoolein cubosomes for the loading and delivery of vitamin B12^7^ and different anticancer compounds, namely, the commonly used 5-fluorouracil,^8,9^ the plant extract isofuranodiene,^10^ and phosphane gold(I) compounds,^11^ and demonstrated that encapsulation increased the drug stability and hence its anticancer activity. Bicontinuous cubic phases consist of a single, continuous bilayer draped over an infinite periodic minimal surface, subdividing space into two interpenetrating but unconnected water networks.^12^ Three types of reversed bicontinuous cubic phases have been identified based on the Schwarz diamond (D, *Pn*3*m*), primitive (P, *Im*3*m*), and Schoen gyroid (G, *Ia*3*d*) minimal surfaces. These systems have the advantage that their physical properties are susceptible to changes in water content, addition of surfactants, temperature, and pressure. In the 2D inverted hexagonal phase, the aqueous domains are composed of densely packed, infinitely long, and straight water-filled rods, embedded in a non-polar matrix composed of lipophilic chains. Even if the reverse hexagonal phase is characterized by a much simpler internal structure than the bicontinuous cubic ones, it has the advantage of better sustaining the release of water-soluble active substances; moreover, its low viscosity is favorable for practical applications.^13^

The possibility of a certain amphiphilic molecule for forming an inverted structure is suggested by its critical packing parameter CPP = *V*/*Al*, where *V* and *l* are the volume and the length of the hydrophobic part of the amphiphile, respectively, and *A* is the cross-sectional area of the hydrophilic headgroup.^14^ Amphiphiles with CPP > 1, characterized by a large hydrophobic portion compared to the polar one, self-assemble into inverse phases with negative curvatures such as the cubic and H_II_ ones. Typically, glyceryl monooleate (monoolein, GMO) as well as other glycerides, such as glyceryl monolinoleate and PHYT, all characterized by a relatively small polar headgroup and a voluminous hydrophobic tail (CPP > 1), form a bicontinuous cubic phase in excess of water at room temperature and transform into a H_II_ phase at higher temperatures. Hence, to exploit the potential of the reversed hexagonal phase, the temperature of the transition from cubic to H_II_ has to be decreased to the one *in vivo*, and this can be obtained upon solubilization of proper additives into the lipid and aqueous domains, such as decane or tricaprylin (hydrophobic), polyethylene glycol (amphiphilic), and sucrose (hydrophilic).^15^ This transition may also be induced, both in PHYT and GMO cubic phases,^16−22^ by incorporation of the hydrophobic guest co-surfactant oleic acid (OA), which determines dehydration of the polar headgroup with a simultaneous increase in the acyl chain volume.

The interest in bicontinuous cubic and reverse hexagonal mesophases for the delivery of therapeutic proteins and peptides is rapidly growing. In fact, the peculiar amphiphilic nature of these systems allows them to accommodate and protect (from enzymatic, physical, and chemical degradation) hydrophilic proteins within the aqueous channel compartment, hydrophobic proteins within the lipid bilayer, and amphiphilic proteins at the interface. 2D and 3D bilayer matrices provide a diffusion pathway for sustained and controlled release due to their large surface areas and specific and controllable water channel sizes.^12^ Protein incorporation in these systems could trigger structural effects that have to be characterized for the dual purpose of optimizing the protein efficiency (*e.g.*, for delivery) and maximizing the protein loading for a specific application. Different hydrophilic proteins, ranging from small cytochrome *c* and lysozyme to large fibrinogen and apo-ferritin, have been incorporated within the lipidic cubic^12^ or hexagonal mesophases,^23^ sometimes inducing structural transitions. Among these proteins is insulin, a protein hormone important for the regulation of glucose metabolism and used worldwide in the treatment of diabetes. The interest in encapsulating this protein in a lipid matrix is due to the need for alternative routes of administration (conventional subcutaneous injection presents several side effects, *e.g.*, pain, irritation, allergy, sometimes hypoglycemia, and lipodystrophy^24^) and solutions to intrinsic stability problems that challenge its formulation and long-term storage. Insulin exists as a monomer, dimer, or hexamer, with the monomer being the biologically active form but, at the same time, being most prone to misfolding and aggregation. After dissociation of the native and thermodynamically stable dimeric form of insulin, the obtained native monomer is metastable and in equilibrium with the amyloid-competent partially folded intermediate, which is favored by low pH and high temperature.^25−27^ The exposure of hydrophobic residues in the partially folded state is the driving force for fibril formation. The hydrophobic clusters (identified as the B-chain segments LVEALYL) tend to establish intermolecular hydrophobic interactions, inducing their secondary structure to switch from α-helix to β-strand and thus forming the nucleus of the insulin fibril.^27^

In previous studies, GMO cubic structures were used to protect insulin from aggregation induced by either agitation,^28^ temperature,^26^ or enzymatic degradation.^29^ On the other hand, different modified reverse hexagonal systems based on GMO and co-surfactants were used by Garti and co-workers to explore the effect of confinement on the stability, morphology, and unfolding behavior of the protein upon heating or pH changes.^30−33^

Herein, we report the encapsulation of insulin in a GMO cubic phase and in a GMO/OA reverse hexagonal phase at low pH values: under these pH conditions, the insulin is in its bioactive (and more unstable) form, whereas the GMO/OA system exhibits a H_II_ structure. The effect of OA incorporation on the structural properties of the GMO matrix was investigated by small angle X-ray scattering (SAXS) and supplemented with the rheological characterization of the prepared systems. Then, the effect of insulin loading on the structural and rheological properties of the systems was analyzed and correlated with the conformational modifications of the protein induced by the confinement and thermal stress, as evidenced by attenuated total reflection-Fourier transform infrared (ATR-FTIR) spectroscopy.

## Experimental Section

### Materials

GMO (Monomuls 90-O18) was kindly provided by BASF, Germany; it has a similar composition to other commercial GMO-based products.^34^ Insulin human recombinant and OA were purchased from Sigma-Aldrich. All solutions were prepared with Milli-Q water. Hydrochloric acid, sodium hydroxide, and chloroform were obtained from Sigma-Aldrich and used without further purification. D_2_O (D, 99.9%), NaOD solution (D, 99%), and DCl (D, 99%) were purchased from Sigma-Aldrich.

### Preparation of LLC Mesophases

Bulk LLC phases were prepared by co-dissolving 40 mg of GMO and OA at three different concentrations (0, 10, and 25 mol %) in chloroform. The solvent was evaporated under a gentle stream of nitrogen, and the mixtures were further dried under vacuum. Excess of water (typically 80% w/w), acidified to pH 3 with HCl, was added to hydrate the samples, which were vortexed and left equilibrating at room temperature for 24 h to form the cubic or hexagonal phase.

Insulin-loaded samples were prepared in the same way but by replacing water in the hydration step with the same volume of a 10 mg/mL insulin aqueous solution (pH, 3): a 4% w/w insulin/lipid ratio was thus obtained.

For ATR-FTIR measurements, 10 mg/mL D_2_O (pD, 3) insulin solution was prepared and used for measurements both on native insulin and insulin-loaded LLC phases. Blank LLC phases were hydrated with D_2_O (pD, 3). The pD was adjusted with DCl or NaOD.

### SAXS Measurements

SAXS measurements were performed at the SAXS beamline of Elettra Sincrotrone Trieste (Trieste, Italy). The beam wavelength was λ = 1.54 Å (8 keV). 2D diffraction patterns were recorded by a Dectris Pilatus 1M placed at 1279 mm from the sample; a vacuum chamber was placed between the sample and the detector to avoid air scattering. The used setup covered the *q* range from about 0.15 to 5 nm^–1^ (*q* = 4π sin ϑ/λ, with 2θ being the scattering angle). Temperature scans ranged between 25 and 70 °C (±0.1 °C), varying the temperature at 1 °C/min rate.

### Rheological Measurements

Rheological analyses were performed using a rotational rheometer (Kinexus Lab+, Malvern) equipped with a 20 mm plate geometry at a gap of 1 mm. Temperature sweep tests were conducted at a frequency of 1 Hz and a stress of 0.5 Pa between 25 and 120 °C at a rate of 1 °C/min. Frequency sweep tests were performed at a shear stress of 0.5 Pa in the frequency range 0.01–10 Hz at the temperatures of 25, 37, 50, 70, and 90 °C.

### ATR-FTIR Measurements

ATR-FTIR measurements were carried out at the IR SISSI beamline, Elettra Sincrotrone Trieste (Trieste, Italy), by using the MIRacle Single Reflection ATR box (PIKE Technologies) with the diamond crystal, mounted on the Vertex 70 interferometer (Bruker Optics) equipped with a deuterated triglycine sulfate detector. Before IR measurements, samples were maintained at 25, 45, 55, and 70 °C for 15 min (native insulin was kept at 70 °C for 3 h). Then, samples were deposited onto the diamond crystal (thermostated at the same temperature of the samples) and purged with a continuous stream of nitrogen gas. ATR-FTIR spectra were collected every 5 s until sample gets dehydrated, as indicated by vanishing of the bands below the detection limit of the combination band of bending and liberation water modes centered at ∼2100 cm^–1^. Each spectrum was acquired in the 4000–550 cm^–1^ spectral range and averaged over 128 scans. A spectral resolution of 4 cm^–1^ was applied. Before each sample acquisition, the background spectrum was collected on the clean diamond crystal, under the same conditions. Raw spectra were corrected for carbon dioxide and water vapor and then vector-normalized in the entire spectral range of acquisition using the atmospheric compensation and vector normalization routines (OPUS 7.5 software, Bruker Optics, Ettlingen, Germany). For each sample, the average absorbance spectrum and the corresponding standard deviation spectra (average absorbance spectrum ± standard deviation spectra) were calculated after 15 min at 25, 45, 55, and 70 °C (for insulin, spectra were also obtained after 3 h at 70 °C). These spectra were curve-fitted in the 1800–1400 cm^–1^ spectral range; the number and the position of the underlying bands were identified by second derivative minima analysis and fixed during the peak-fitting procedure with Gaussian functions; the integrated areas of all the underlying bands were obtained (GRAMS/AI 9.1, Galactic Industries, Inc., Salem, New Hampshire).

## Results and Discussion

**Figure 1:**
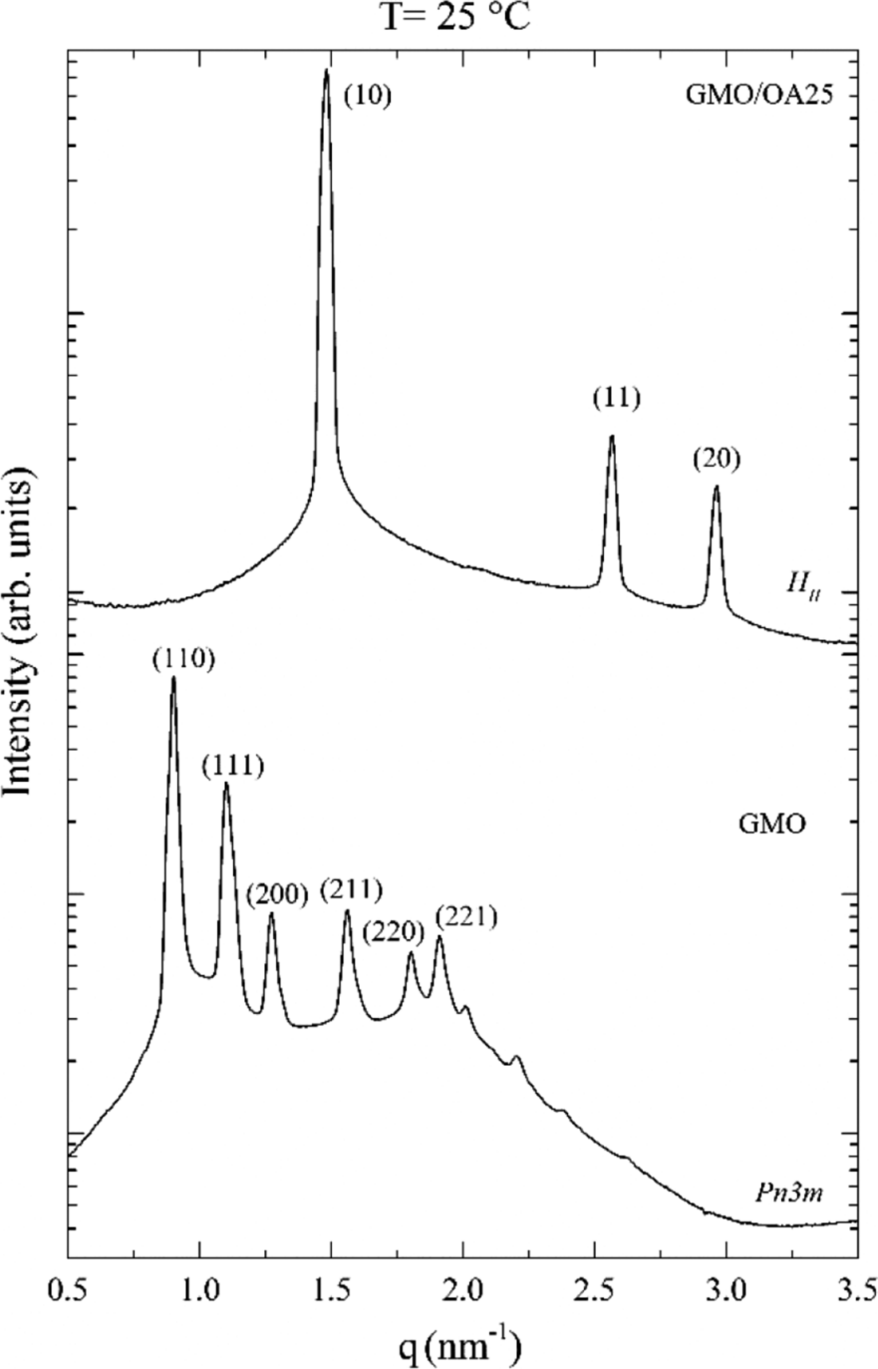
Representative SAXS patterns for GMO and GMO/OA 25% at 25 °C. Peak indexing indicates a *Pn*3*m* and a H_II_ mesophase, respectively.

Liquid crystalline GMO and GMO/OA systems (empty and loaded with insulin) were characterized by SAXS, rheological, and ATR-FTIR measurements. SAXS was used to determine the symmetry and geometric parameters of the liquid crystalline systems as functions of OA content and temperature and to evaluate the impact of insulin encapsulation on these structures. Indexing the SAXS diffraction patterns reveals that the GMO empty matrix, in excess of water at pH = 3 and 25 °C, assembles in a *Pn*3*m* cubic phase with a lattice parameter of 9.84 nm, whereas the addition of OA (10 and 25 mol %) induces the transition to hexagonal phases H_II_ with lattice parameters of 5.81 and 4.91 nm, respectively. Representative SAXS patterns, showing the characteristic peak sequence of the two mesophases, are presented in Figure 1.

The effect of OA on the GMO/OA phase behavior is pH-dependent^35^ and correlated to the protonation/deprotonation of OA headgroup at different pHs. The observed *Pn*3*m* to H_II_ transition is well documented in the literature^18,35^ and is derived from the fact that at low pHs the OA carboxyl group is in a protonated form (p*K*_a_, OA ∼ 5), thus lowering the surface charge density and the polarity of the membrane interface. It follows that the number of water molecules interacting with the headgroup decreases, that the cross-sectional area of the hydrophilic headgroup is smaller for GMO/OA than for GMO, and hence the CPP value for the GMO/OA system is not only larger than 1 but likely also larger than the corresponding value for GMO. In fact, the CPP = *V*/*Al*^14^ (with *V* and *l* being the volume and the length of the hydrophobic part of the amphiphile, respectively, and *A* the cross-sectional area of the hydrophilic headgroup) is larger than 1 for reverse structures and increases in the case of a more negatively curved mesophase, such as the hexagonal one. Table 1 summarizes the lattice parameter *a* for all the studied systems together with the corresponding water channel radius, calculated by using the relation *r*_w_ = [(−σ/2πχ)^1/2^*a*] – *l* where *l* is the lipid length^36^ and σ and χ are the topological constant characteristics of a given cubic phase (*Pn*3*m* structures σ = 1.919 and χ = −2). It can be observed that, at 25 °C, both the parameters decrease upon the addition of increasing amount of OA.^19^

**Table 1:** Phase Structure and Lattice Parameters of GMO, GMO/OA 10%, and GMO/OA 25% Systems, Empty and Insulin (Ins)-Loaded, as a Function of Temperature

	*T* = 25 °C		*T* = 45 °C		*T* = 55 °C		*T* = 70 °C	
		Ins 4% w/w		Ins 4% w/w		Ins 4% w/w		Ins 4% w/w
GMO	*Pn*3*m*	*Pn*3*m*	*Pn*3*m*	*Pn*3*m*	*Pn*3*m*	*Pn*3*m*	*Pn*3*m*	*Pn*3*m*
	*a* = 9.84 nm	*a* = 9.83 nm	*a* = 8.78 nm	*a* = 8.81 nm	*a* = 8.18 nm	*a* = 8.21 nm	*a* = 7.59 nm	*a* = 7.61 nm
	*r*_w_ = 2.12 nm	*r*_w_ = 2.12 nm	*r*_w_ = 1.78 nm	*r*_w_ = 1.79 nm	*r*_w_ = 1.58 nm	*r*_w_ = 1.59 nm	*r*_w_ = 1.40 nm	*r*_w_ = 1.40 nm
GMO/OA 10 mol %	H_II_	H_II_	H_II_	H_II_	H_II_	H_II_	H_II_	H_II_
	*a* = 5.81 nm	*a* = 6.19 nm	*a* = 5.45 nm	*a* = 5.79 nm	*a* = 5.31 nm	*a* = 5.60 nm	*a* = 5.1 nm	*a* = 5.35 nm
	*r*_w_ = 1.17 nm	*r*_w_ = 1.36 nm	*r*_w_ = 1.07 nm	*r*_w_ = 1.25 nm	*r*_w_ = 1.03 nm	*r*_w_ = 1.18 nm	*r*_w_ = 0.98 nm	*r*_w_ = 1.10 nm
GMO/OA 25 mol %	H_II_	H_II_	H_II_	H_II_	H_II_	H_II_	*L*_2_	H_II_
	*a* = 4.91 nm	*a* = 5.20 nm	*a* = 4.69 nm	*a* = 4.93 nm	*a* = 4.6 nm	*a* = 4.81 nm	3.9 nm	*a* = 4.65 nm
	*r*_w_ = 0.72 nm	*r*_w_ = 0.87 nm	*r*_w_ = 0.69 nm	*r*_w_ = 0.81 nm	*r*_w_ = 0.68 nm	*r*_w_ = 0.78 nm		*r*_w_ = 0.75 nm

**Figure 2:**
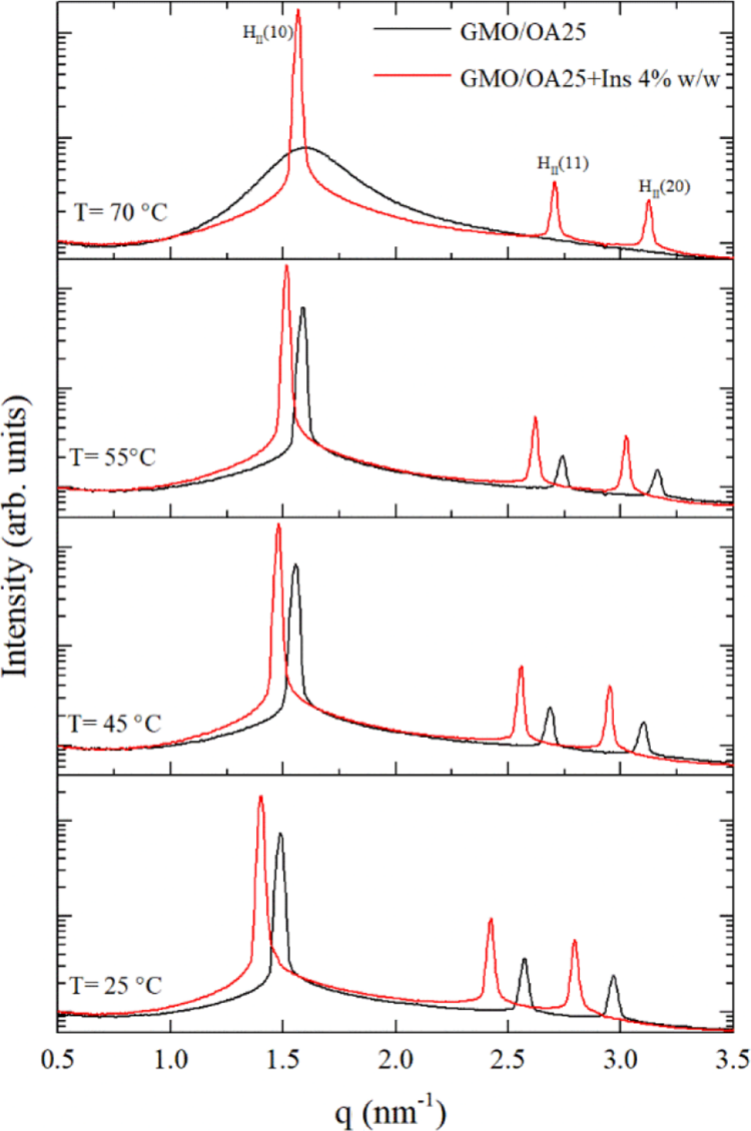
Representative SAXS patterns for GMO/OA 25% and GMO/OA 25% + Ins 4% as a function of temperature.

On heating, the lattice parameter slightly decreases for all the systems: for the GMO *Pn*3*m* phase, from 9.84 nm (25 °C) to 7.59 nm (70 °C); for the GMO/OA 10% H_II_ phase, from 5.81 nm (25 °C) to 5.1 nm (70 °C), and for the GMO/OA 25% H_II_ phase, from 4.91 nm (25 °C) to 4.5 nm (68 °C). For the latter system, a micellar *L*_2_ phase was observed at 70 °C (Figure 2 and Table 1). This trend can be justified either by dehydration of the polar headgroups, resulting in an increase of the interfacial curvature, or by a greater disorder of the lipid hydrocarbon chains, which become more fluid with increasing temperature, thus determining the decrease of bilayer thickness.^36,37^

At 25 °C, insulin encapsulation (4% w/w) in the three lipid matrices had no effect on the structural symmetry with respect to the empty ones, as also observed by Strachan *et al.*(29) and Kraineva *et al.*(26) (note that in the latter case, the cubic phase considered was an *Ia*3*d* instead of a *Pn*3*m* because of the different hydration conditions of GMO, 20% *vs* excess of water). In the loaded lipid matrices, a very slight decrease of the cell unit and the aqueous radius channel was observed for GMO, whereas the opposite effect was observed in the presence of OA, as shown in Figure 2 and Table 1. These results can be justified comparing the different water channel radii of the two kinds of mesophases with the radius of gyration of the protein, *ca.* 1.49 nm for the dimer;^26^ the favorite insulin forms at a low concentration over the pH range 2–8, and 1.16 nm for the monomer. Thus, the size of the water channels in the *Pn*3*m* mesophase is compatible with the size of both the monomer and the dimer, and almost no differences were observed in the two parameters in the presence and absence of insulin. With the H_II_ mesophase, the radii of the cylinders are smaller than insulin dimensions, and its incorporation resulted in an increase of the lattice parameter. In fact, insulin accommodation in the small aqueous cylinders of the hexagonal mesophase (1.17 and 0.72 nm for GMO/OA 10% and GMO/OA 25%, respectively) results in a considerable strain of the hexagonal lattice, which is released by the swelling of the system.^29^ In fact, the protein has to be partially intercalated in the interface region to fit in the water cylinders, and this embedment determines an increase in the lattice parameter of the structure, as actually observed in the SAXS measurements.^38^ Moreover, these changes may derive from the chaotropic effect of insulin, which determines a destabilization of the structure of bulk water in the aqueous cylinders and the consequent accumulation of the protein at the lipid–water interface.^13^

The phase behavior of the three insulin-loaded systems was studied as a function of the temperature (Table 1): the evolution of SAXS patterns for GMO/OA 25%, empty and loaded, is compared in Figure 2. As observed with the corresponding empty systems, the lattice parameter slightly decreased with temperature, in accordance with the fact that the increasing temperature determines both the dehydration of the polar headgroups and the increase of the lipid hydrocarbon chain fluidity. An exception is represented by GMO/OA 25%: in this case, a phase transition from the H_II_ to *L*_2_ phase was observed in the empty system at 70 °C; by contrast, the presence of insulin stabilized the H_II_ phase, which persisted over the whole temperature range with a decreasing lattice parameter.

**Figure 3:**
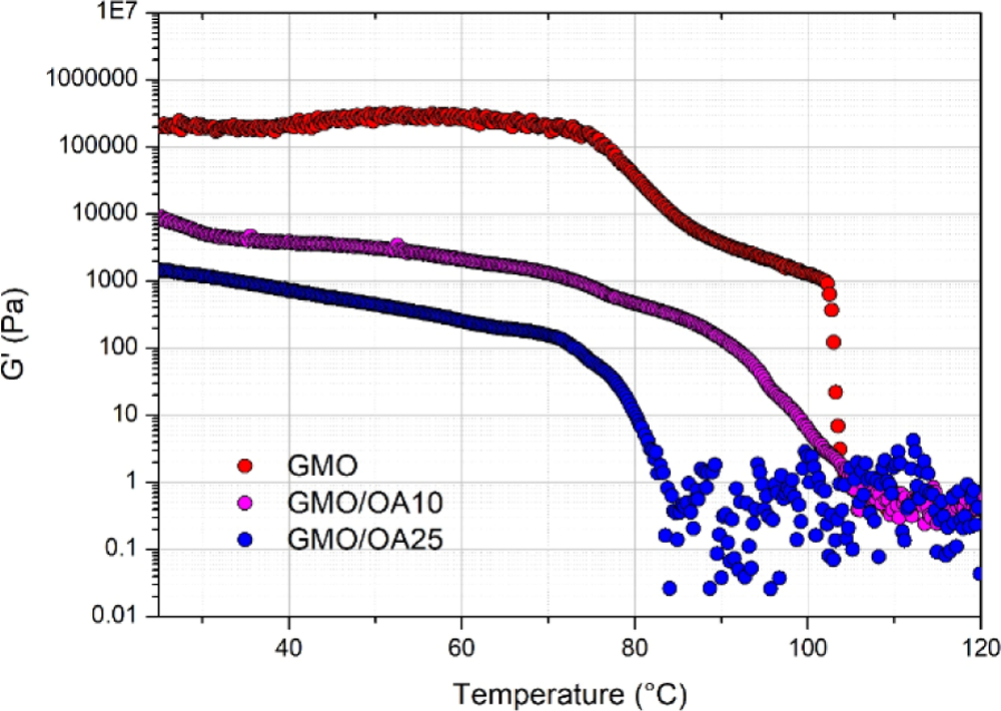
Variation of the elastic modulus (*G*′) as a function of temperature for GMO, GMO/OA 10%, and GMO/OA 25% (temperature sweep test).

The viscoelastic properties of different GMO-based systems (empty and insulin-loaded) were investigated using oscillatory rheological measurements by monitoring the variation of the rheological moduli (*G*′ and *G*″) as a function of the frequency (frequency sweep test) or temperature (temperature sweep test). The variation of the elastic modulus (*G*′) with temperature, for GMO alone and in combination with 10 and 25% of OA, is shown in Figure 3.

As reported for other liquid crystalline phases,^39^ the temperature sweep test can highlight the variation in the rheological properties of the sample derived from the changes in mesophases as a function of temperature. Indeed, the elastic modulus (*G*′) for all analyzed samples showed a marked temperature dependency. Specifically, the decrease of *G*′ with temperature was not linear, indicating that samples underwent thermal transitions involving a decrease in their consistency (*e.g.*, from cubic to hexagonal phases) in the 25–120 °C temperature range. The *G*′ profile of GMO over temperature is similar to that obtained for monolinolein, as reported in the literature.^39^ Between 25 and 70 °C, the relatively constant high value of *G*′ (>100 000 Pa) suggests the presence of a *Pn*3*m* cubic phase, in accordance with SAXS measurements. At 70 °C, a sharp decrease in *G*′ was observed, likely ascribable to the onset of the conversion of the cubic phase into the hexagonal phase. GMO hexagonal phases exist up to around 100 °C, above which a further decrease in viscosity indicates the complete melting and appearance of a liquid phase (*L*_2_ + water). This is supported by considering the variation of the phase angle parameter from the temperature sweep test. Indeed, phase angle values remain below 45° (solid-like behavior) in the temperature range in which cubic and hexagonal phases exist (Figure S1).

In the presence of OA, the consistency of the systems was lower than that for pure GMO over the whole temperature range, with the strongest reduction observed for the largest OA concentration (25%). This is due to the GMO/OA samples being in the hexagonal phase already at room temperature, as also evidenced by SAXS measurements. Moreover, differently from GMO, the rheological modulus showed a constant decrease from 25 °C to the temperature at which samples become liquid-like, indicating a stronger sensitivity to the temperature of the H_II_ phase in comparison to that of the *Pn*3*m* phase. The temperature at which a liquid phase formed seems to be dependent on the OA concentration, being more than 100 °C for GMO/OA 10%, and about 85 °C for GMO/OA 25%. Also, this result is in agreement with SAXS data, showing the appearance of a *L*_2_ phase at 70 °C for GMO/OA 25% but not for GMO/OA 10%.

**Figure 4:**
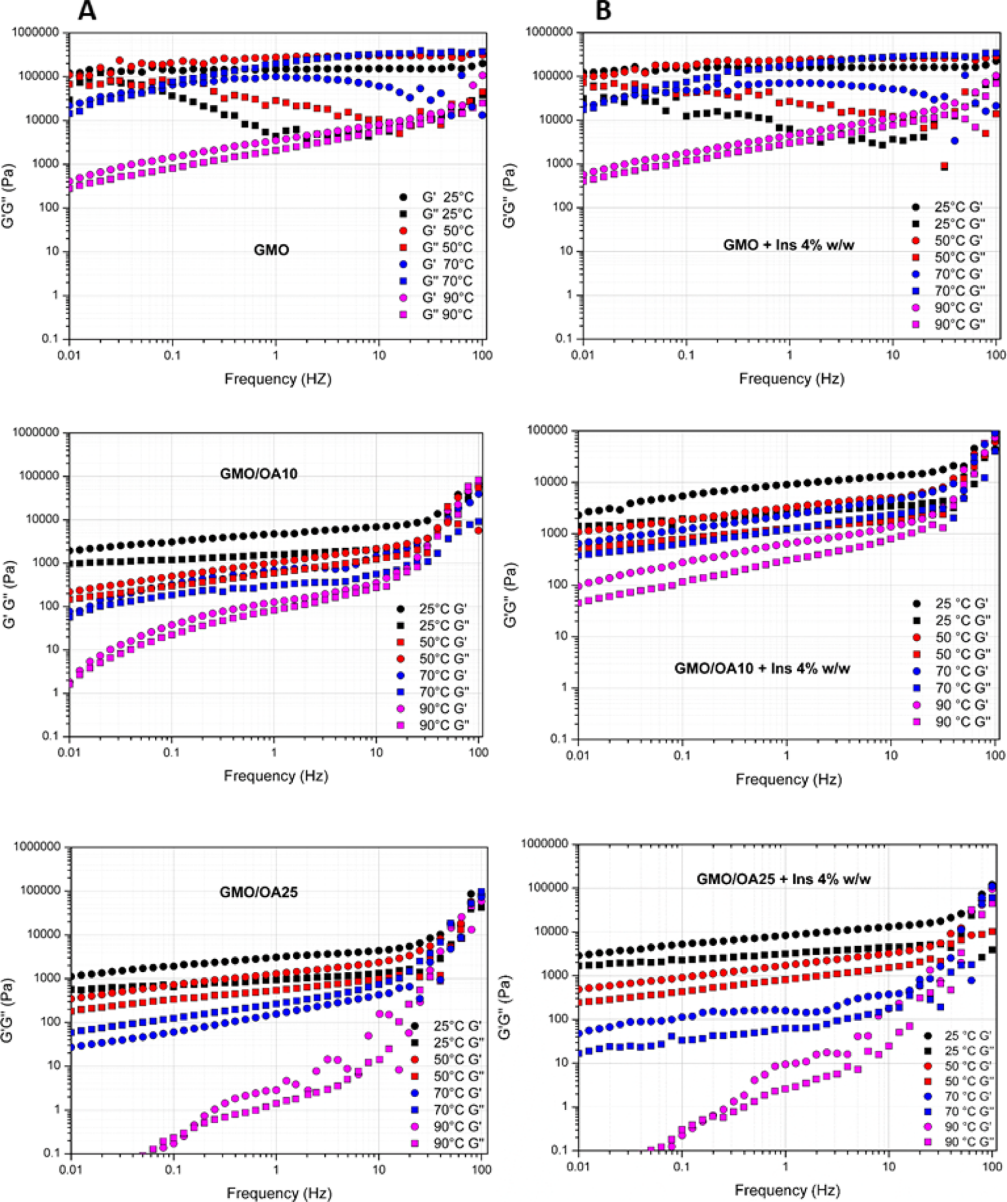
Variation of the elastic (*G*′, circle) and viscous (*G*″, square) moduli over frequency (0.01–10 Hz) for (A) GMO, GMO/OA 10%, and GMO/OA 25% and (B) GMO + Ins 4%, GMO/OA 10% + Ins 4%, and GMO/OA 25% + Ins 4% at different temperatures (25, 50, 70, and 90 °C).

Frequency sweep tests were carried out to further elucidate the viscoelastic properties of GMO or GMO/OA systems, since the internal arrangements of GMO molecules in the various mesophases have a different rheological response to the applied frequency when analyzed in the linear viscoelastic regimen. To this purpose, based on the SAXS measurements and the temperature sweep tests, frequency measurements were performed at temperatures of 25, 50, 70, and 90 °C. The results are reported in Figure 4A.

As previously reported,^40^ the cubic phase is the most rigid of the GMO mesophases, displaying a solid-like prevalent behavior with *G*′ higher than *G*″ modulus in the middle–high range of frequencies (0.1–100 Hz) (Voigt behavior), and a Maxwell liquid behavior at lower frequencies (<0.1 Hz) (from a rubbery plateau to the transition-to-flow regions according to the model of an ideal viscoelastic material). For GMO at 25 and 50 °C, the trend of *G*′ and *G*″ moduli confirmed the presence of a cubic phase. Specifically, *G*′ is essentially independent of the applied frequency, while *G*″ shows an increment at high frequency (>10 Hz), which is the typical trend described by the “slip plane” model developed for cubic phases.^41^ According to this theory, above a critical applied stress, the cubic phases can be represented as the crystalline “slip planes” of cooperatively diffusing molecules, which have a bulk relaxation mechanism. The topology of the internal network is maintained since the deformation can be explained by the flow of layers rather than involving a real “breaking and forming” of bonds at the edge of the cubic unit cell.

A decrease in the rigidity was determined by the transition from the cubic *Pn*3*m* to the hexagonal phase, as evidenced by the lower values of the rheological moduli *G*′ and *G*″. At 70 °C, a temperature at which the two phases co-exist, an intermediate behavior between the two mesophases was observed. At this temperature, the *G*′ modulus is significantly dependent on frequency, whereas it becomes almost frequency-independent at lower temperatures. At 90 °C, a marked dependency on the frequency of both rheological moduli was observed that is typical for the GMO hexagonal phase acting as a viscoelastic fluid with a prevalent liquid-like behavior at low frequencies and a prevalent solid-like behavior at higher frequencies. Generally, samples with this rheological behavior show cross-over frequencies of the rheological moduli in the mid-range of the viscoelastic spectrum, resulting in a large dissipation of energy of the material upon solicitation in shorter times than cubic phases. For the tested samples, these cross-over points were not always observed in the range of frequencies analyzed. Indeed, a cross-over point was observed at about 0.05 Hz for GMO at 70 °C, whereas it was shifted at lower frequencies for GMO/OA hexagonal phases. The observed monotonic increase of *G*′ and *G*″ in a relatively large range of frequencies can be ascribed to the properties of hexagonal phases to align in the direction of the applied stress at different frequencies, as commonly observed for dilute polymeric dispersions.^42^

The decrease in consistency at any analyzed temperature in GMO/OA was also evident from the frequency sweep test, confirming the ability of OA to stabilize GMO in a hexagonal mesophase, also at temperatures at which GMO typically self-assembles into a cubic phase. Indeed, GMO/OA 25% has the lowest values for both the elastic modulus (*G*′) and viscous modulus (*G*″) at any frequency and temperature. Particularly, in the case of GMO/OA 25% at 90 °C, the formation of a micellar *L*_2_ + water phase was confirmed by the low values for *G*′ and *G*″, together with the marked dependency on frequency and the prevalence of the elastic modulus over the viscous one.

**Figure 5:**
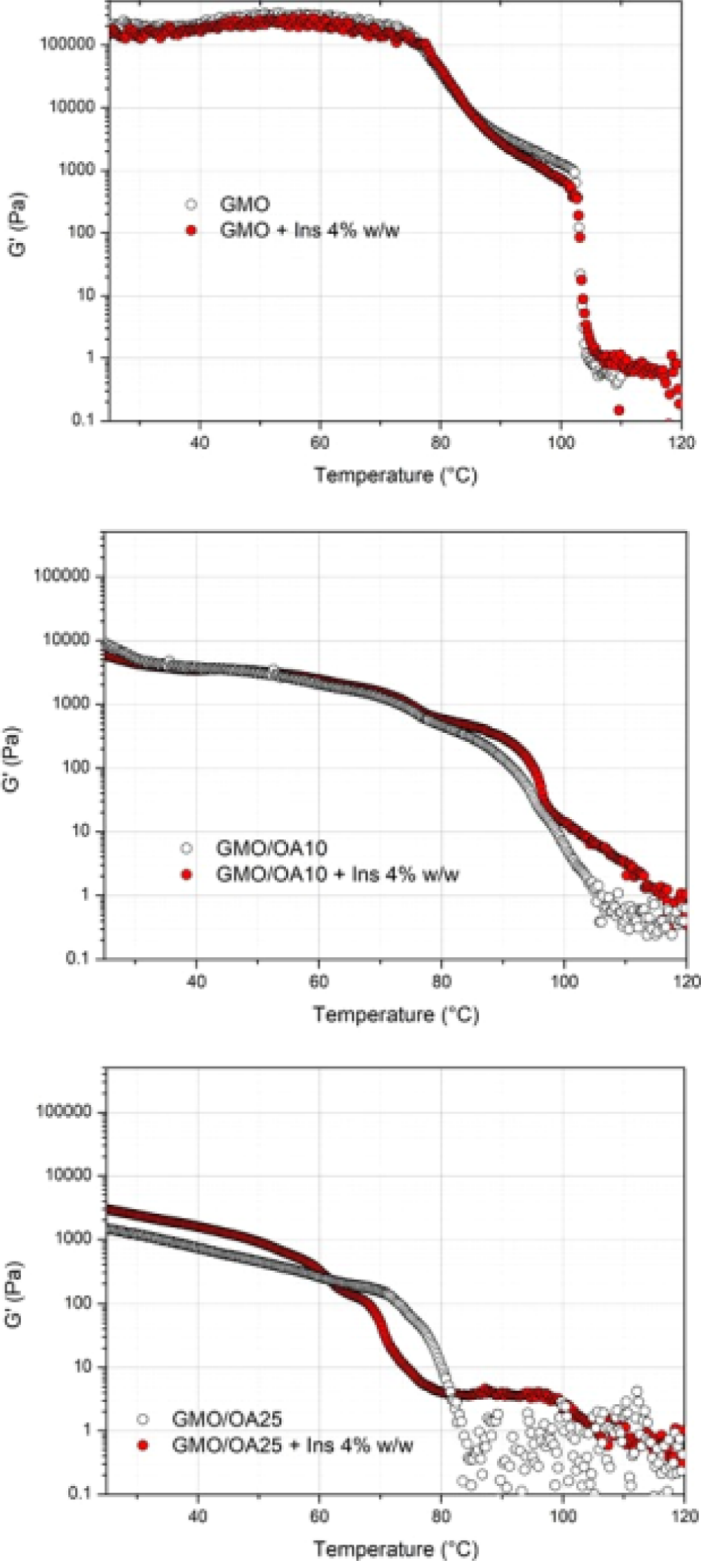
Variation of the elastic modulus (*G*′) as a function of temperature for GMO, GMO/OA 10%, and GMO/OA 25%, both unloaded and loaded with 4% of insulin (temperature sweep test).

Temperature sweep tests were also useful in highlighting the effect of the incorporation of insulin on the phase transition of GMO and GMO/OA (Figure 5). Actually, the presence of insulin influenced the consistency of the sample only in the presence of OA. In fact, in GMO + Ins, no appreciable differences were observed in the *G*′ trend with temperature compared to that of the empty GMO. In GMO/OA 10%, the effect of insulin encapsulation was appreciable only at temperatures >80 °C, while in GMO/OA 25% some differences in the *G*′ values were evident at all analyzed temperatures. Specifically, *G*′ values were slightly larger for the loaded GMO/OA 25% than for the corresponding empty sample below 60 and above 80 °C, temperatures at which only one phase is present, hexagonal or lamellar, respectively. In the 60–80 °C range, in which the transition from the hexagonal to lamellar phase occurs, *G*′ values are slightly lower. The differences in the consistency of the samples (GMO/OA) in the presence of insulin can be related to the effect of the peptide in increasing the lattice parameters, especially in the case of the hexagonal phases, as reported in Table 1. The water channels, whose radii decreased upon the addition of increasing amount of OA, were too narrow to host insulin inside them, hence insulin had to intercalate in the interface region enhancing the lattice parameter (as demonstrated by SAXS experiments). The presence of insulin determined a further dehydration of the overall systems, promoting the hydrophobic interactions and resulting in an increase of the consistency and stabilization of the hexagonal mesophase.

The presence of insulin determined only little variations in the frequency dependence of the rheological moduli at different temperatures (Figure 4B). The effect of protein incorporation was more evident on the hexagonal phase of GMO, rather than on the cubic one, and particularly in the presence of OA. In insulin-loaded GMO/OA, the elasticity of the hexagonal phase increased, likely because of the formation of more stabilized hydrophilic interactions at the lipid–water interface within the cylindrical structures forming the lattice, as also reported in ref (33). In GMO/OA 25% at temperature >50 °C, the presence of insulin determines a decrease in *G*″ values and a weaker dependency of *G*′ on frequency, indicating a more elastic behavior.

ATR–FTIR spectroscopy was exploited to investigate the modifications occurring in the secondary structure of insulin, both native and loaded in GMO and GMO/OA matrices, as a function of the temperature. Amide I and II are convoluted bands centered respectively at ∼1650 and ∼1540 cm^–1^, which arise from the vibrational modes of the peptide linkage; they are diagnostic to evaluate modifications in proteins’ secondary structure, since the position of their underlying bands (in terms of wavenumbers) is strictly influenced by hydrogen bonding. In particular, amide I/I′ is due to the stretching vibration of C=O (ν_C=O_, 76%) and C–N (ν_C–N_, 14%) and the bending vibration of N–H (ν_N–H_, 10%), whereas amide II/II′ is attributed to the bending vibration of N–H (ν_N–H_, 60%) and the stretching vibration of C–N (ν_C–N_, 40%).^43^

**Figure 6:**
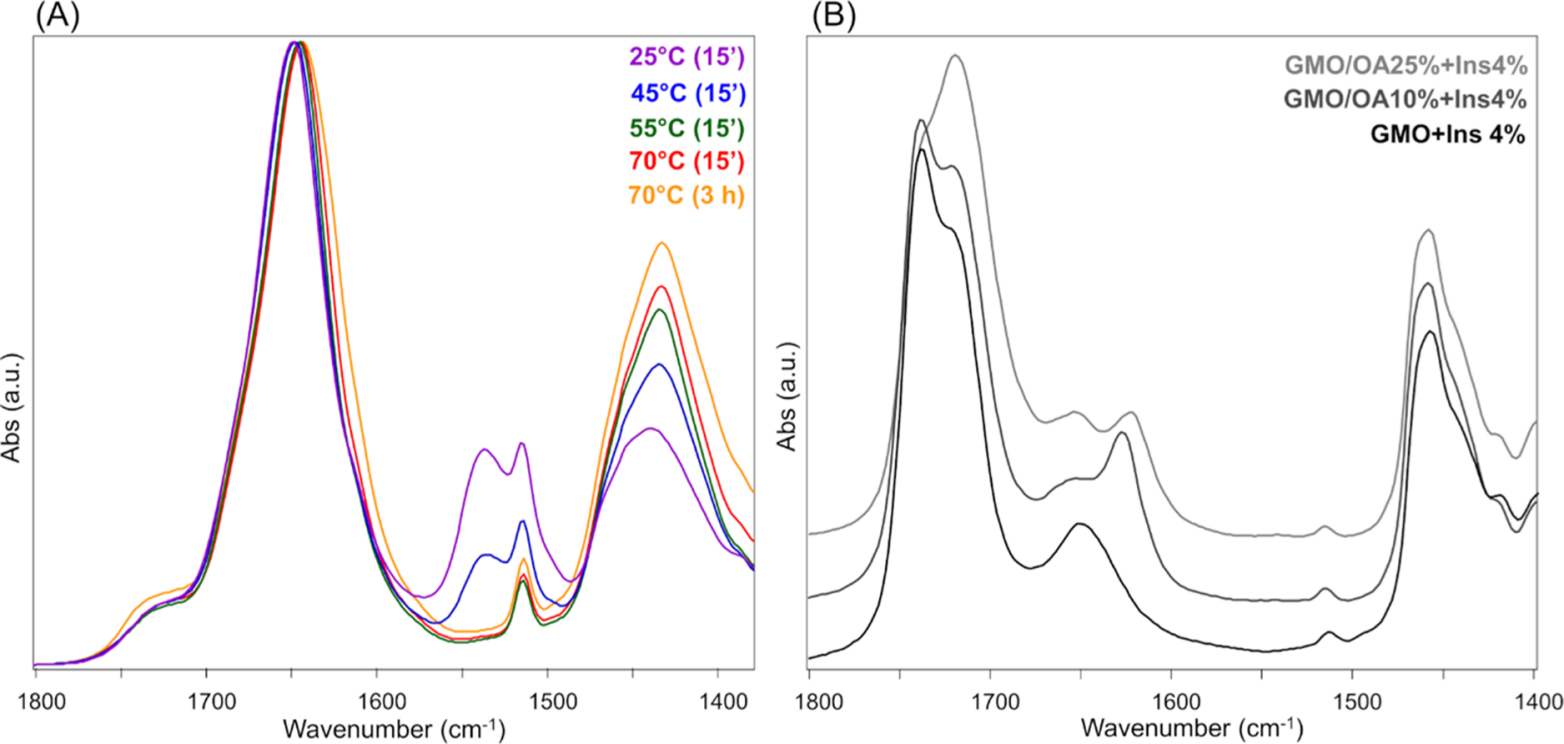
(A) Absorbance ATR-FTIR spectra of insulin in D_2_O solution at pD = 3; spectra were acquired after 15 min at 25 °C (violet), 45 °C (blue), 55 °C (green), 70 °C (red), and after 3 h at 70 °C (orange) (1800–1380 cm^–1^ spectral range). (B) Absorbance ATR-FTIR spectra of GMO + Ins 4% (black), GMO/OA 10% + Ins 4% (dark gray), and GMO/OA 25% + Ins 4% (light gray) systems acquired after 15 min at 70 °C. Spectra are reported in the 1800–1400 cm^–1^ range and are shifted along the *y*-axis for better viewing.

The ATR–FTIR spectra of insulin in D_2_O solution at pD 3, acquired after 15 min at 25, 45, 55, and 70 °C and after 3 h at 70 °C, are shown in Figure 6A: the underlying peaks of amide I/I′ and II/II′ bands, whose positions were determined by peak fitting analysis, are reported in Table 2, together with the corresponding vibrational modes and chemical/structural assignments. At 25 and 45 °C, the amide I/I′ was centered at *ca.* 1648 cm^–1^, while for the amide II, two bands at ∼1540 cm^–1^ (ν_N–H_, mainly) and ∼1514 cm^–1^ (vibrational modes of tyrosine) were detected. After 15 min at 55 and 70 °C, a shift of amide I/I′ to 1642 cm^–1^ was observed; moreover, at the same temperatures, the disappearance of the band of amide II at 1540 cm^–1^ and the contemporary increase of the amide II′ band at 1432 cm^–1^ were evidenced; no changes were found in the band of tyrosine at 1514 cm^–1^. These findings have already been described in the literature^44^ and are indicative of the complete H/D exchange favored by the partial unfolding and dissociation of the dimeric form. No significant differences were found between the insulin spectra collected both after 15 min and 3 h at 70 °C.

**Table 2:** Amide I/I′ and II/II′ Underlying Bands, Calculated by the Fitting Procedure, for Insulin at pD 3 in D_2_O Solution at Different Temperatures

			wavenumber (cm^–1^)				
insulin vibrational modes		structural/chemical assignment	25 °C (15 min)	45 °C (15 min)	55 °C (15 min)	70 °C (15 min)	70 °C (3 h)
amide I/I′	ν_C=O_, δ_N–H_, ν_C–N_	antiparallel β-sheets	1687, 2161	1687, 1612	1685, 1609	1682, 1609	1683, 1608
		α-helices	1662	1660	1657	1655	1655
		random coils	1644	1644	1642	1642	1641
		parallel β-sheets	1627	1627	1628	1627	1627
amide II/II′	δ_N–H_, ν_C–N_	N–H	1540	1540	1540		
	ν_C=C_	tyrosine	1514	1514	1514	1514	1514
	δ_N–D_	N–D		1432	1432	1432	1432

ATR-FTIR spectra of insulin in GMO and GMO/OA matrices were collected after 15 min of incubation at each temperature. Since no significant difference was found in the absorbance profiles of native insulin both after 15 min and 3 h at 70 °C, the latter time point was not considered. Moreover, no changes with temperature were found in the ATR-FTIR spectra of the empty matrices (data not shown).

When insulin was encapsulated in lipid matrices, the obtained IR spectra were prevalently composed by the bands of GMO and GMO/OA (Figure 6B). Nevertheless, some weak signals attributable to amide I/I′ and II/II′ bands of the protein could still be distinguished. In particular, the band centered at *ca.* 1653 cm^–1^, due to the ν_C=C_ of GMO and OA, can also be ascribable to amide I/I′ of insulin and, in order to evidence the effect of the increasing temperature on insulin conformation, we focused on this band and specifically on its fitting as discussed below. Moreover, the tyrosine band at 1514 cm^–1^ is present in all complexes together with a very weak band at 1538 cm^–1^, ascribable to amide II of insulin. In the ATR–FTIR spectra of GMO/OA 10% + Ins 4% and GMO/OA 25% + Ins 4% at 70 °C, a band at 1627 cm^–1^ arises, suggesting the occurrence of β-sheet structures.

**Figure 7:**
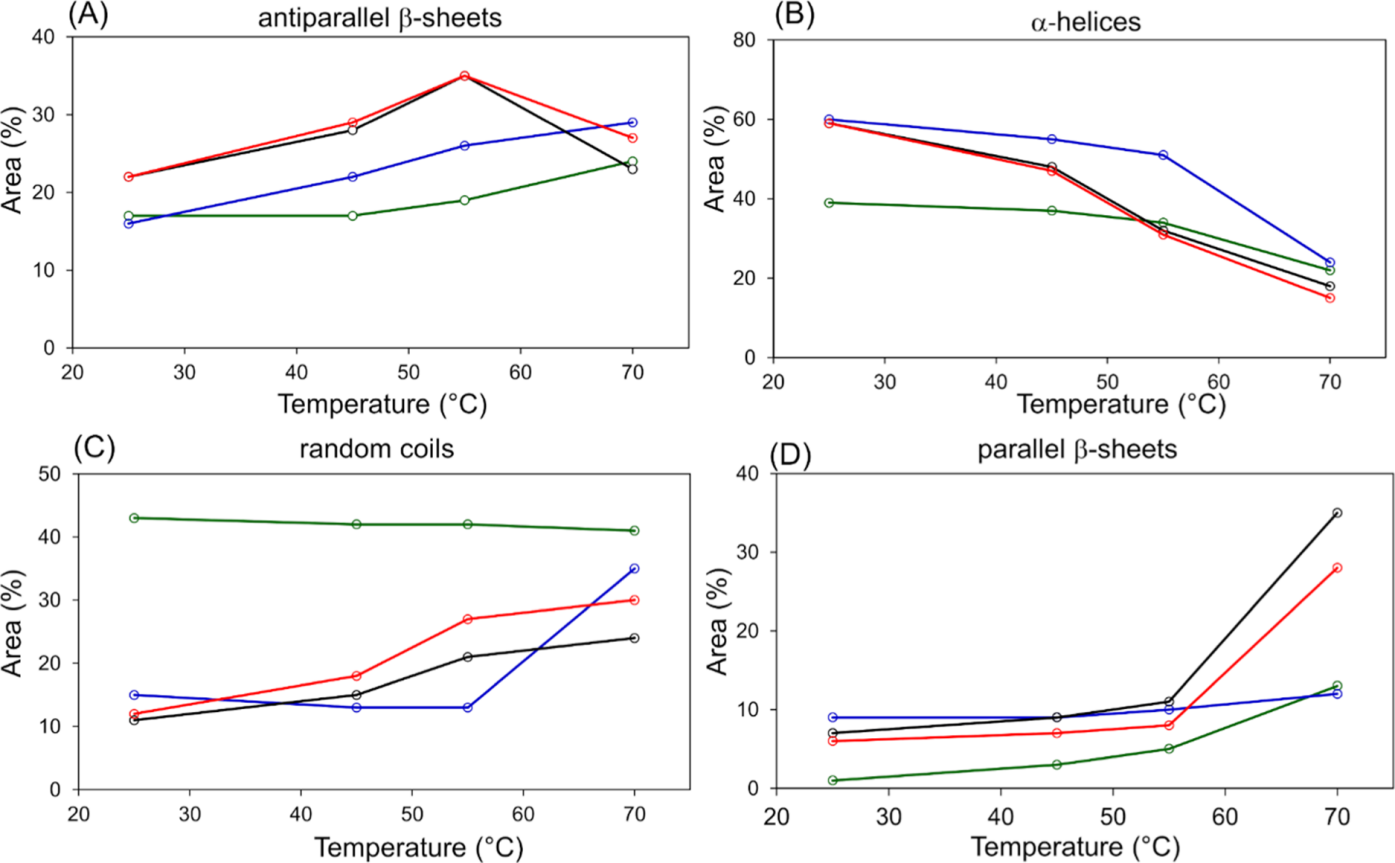
Line graphs representing the percentage areas of antiparallel β-sheets, α-helices, random coils, and parallel β-sheets of the secondary structures calculated for Ins 4% (green), GMO + Ins 4% (blue), GMO/OA 10% + Ins 4% (black), and GMO/OA 25% + Ins 4% (red) at 25, 45, 55, and 70 °C (15 min).

To evaluate the modifications occurring in the secondary structure of insulin related to temperature and lipid matrices, the percentage areas of the antiparallel β-sheets (calculated as the ratio between the sum of the areas of the bands centered at 1687 and 1612 cm^–1^ and the sum of all the underlying bands of amide I//I′), α-helices (calculated as the ratio between the area of the band centered at 1662 cm^–1^ and the sum of all the underlying bands of amide I/I′), random coils (calculated as the ratio between the area of the band centered at 1644 cm^–1^ and the sum of all the underlying bands of amide I/I′), and parallel β-sheets (calculated as the ratio between the area of the band centered at 1627 cm^–1^ and the sum of all the underlying bands of amide I/I′) of the secondary structures were calculated (Table 3) and reported as line graphs in Figure 7.

**Table 3:** Percentage Areas of Secondary Structures of Insulin at pD 3 in D_2_O Solution, Both Native and Loaded in GMO and GMO/OA Matrices at 25, 45, 55, and 70 °C

		percentage areas (%)				
	secondary structures	25 °C (15 min)	45 °C (15 min)	55 °C (15 min)	70 °C (15 min)	70 °C (3 h)
Ins 4%	antiparallel β-sheets	17	17	19	24	27
	α-helices	39	37	34	22	10
	random coils	43	42	42	41	48
	parallel β-sheets	1	3	5	13	15
GMO + Ins 4%	antiparallel β-sheets	16	22	26	29	
	α-helices	60	55	51	24	
	random coils	15	13	13	35	
	parallel β-sheets	9	9	10	12	
GMO + OA 10% + Ins 4%	antiparallel β-sheets	22	28	35	23	
	α-helices	59	48	32	18	
	random coils	11	15	21	24	
	parallel β-sheets	7	9	11	35	
GMO + OA 25% + Ins 4%	antiparallel β-sheets	22	29	35	27	
	α-helices	59	47	31	15	
	random coils	12	18	27	30	
	parallel β-sheets	6	7	8	28	

At 25 °C and pD = 3, native insulin is stable in the dimeric form, and its secondary structure is found to be in good agreement with the X-ray data of insulin obtained at low pH^45^ and with those reported by other authors.^26^ The amide I band is characterized by *ca.* 40% of the α-helix component, as well as by random coils, while the β-sheet conformation (both parallel and antiparallel) accounted for *ca.* 20% (Table 3).

By increasing the temperature, the α-helical structure drops in favor of β-sheets (parallel and antiparallel), which can be considered the nucleus of the insulin fibril, in accordance with the model proposed by Ivanova *et al.*,^27^ and this decrease is more evident after 3 h at this temperature (Table 3).

Upon insulin encapsulation in the lipid matrices, GMO or GMO/OA systems may alter the energy of the heat-induced misfolded state by interfering with the exposure of the hydrophobic residues responsible for the α-helix to β-strand conformational change and by restricting the configurational degrees of freedom in a confined environment.

In GMO + Ins 4%, the α-helix band decreased significantly upon heating with the concomitant increase of the random coils, whereas just a small increase in the β-sheets was observed. According to these results, GMO cubic system seems to favor the heat-induced unfolding toward an unordered structure, rather than to a β-sheet-rich misfolded state, prone to aggregation into fibrils.

The similarity between GMO/OA 10% + Ins 4% and GMO/OA 25% + Ins 4% complexes observed in the spectral profiles is particularly evident in the graphs of Figure 7, where the same trends for the different components of the amide I/I′ bands were observed: the α-helix component decreased, whereas the parallel β-sheet component dramatically increased. In these cases, the co-presence of OA and GMO arranged in a hexagonal phase seems to have an impact on the kind of β-sheets formed in the heat-induced folding intermediate. In fact, we can observe a prevalence of parallel β-sheets as the temperature increases, which can account for a misfolded intermediate behaving as a β-sheet-rich nucleus for fibril formation. It is conceivable that the different polarities and the smaller space available in the hexagonal channels force the C-terminal of the monomeric insulin B-chain to unfold in order to allow a better hosting of the GMO/OA system. This partial unfolding promotes the α-helix to β-strand conversion of both the B-chain segment LVEALYL and the A-chain segment LYQLENY which further pack in the β-sheet-rich spine of the insulin fibril.

## Conclusions

The use of complementary techniques including SAXS, rheological measurements, and ATR-FTIR spectroscopy provided a useful approach for studying the effect of encapsulated insulin on the cubic and hexagonal mesophases and of the confinement in nanostructured water channels on the secondary structure of the protein and hence on its stability. Data collected at room temperature showed that insulin encapsulation in GMO cubic mesophase did not affect the structure since the aqueous channels are large enough to load the protein even in the dimeric form. Conversely, the encapsulation of insulin in GMO/OA hexagonal phases induced an increase of the unit cell dimensions without any modification in the symmetry of the structure, likely because the water channels are smaller than insulin dimensions. Therefore, they cannot host the protein unless an increase in the lattice parameter occurs. As expected, the lattice parameter slightly decreased upon heating up to 70 °C for all the systems (empty or loaded), and only for GMO/OA 25% + Ins 4% at 70 °C, the addition of insulin stabilized the H_II_ phase instead of the *L*_2_ phase, which was observed at this temperature in the empty system.

Rheological analyses (temperature sweep test) confirmed the influence of insulin unfolding above 70 °C, as resulted from the increase of the consistency and stabilization of the hexagonal mesophase, especially occurring in the systems prepared with GMO and OA.

The effect of the confinement into the cubic and hexagonal lipid matrices on the secondary structure of insulin was revealed by the ATR-FTIR spectroscopic data. In both kinds of mesophases, insulin encapsulation determined changes in its components to favor the accommodation/intercalation of the protein in the matrices with a preference for more ordered structures, namely, α-helix and β-sheets, instead of the random coils. Heating from 25 to 70 °C caused a decrease of the insulin α-helix content with a concomitant increase of the random coil structure in the GMO cubic phase and of the parallel β-sheet content in hexagonal phases (GMO/OA 10% and GMO/OA 25%), which favors aggregation and fibril formation.

From this study about protein encapsulation within cubic and hexagonal phases at different temperatures, useful information was obtained which could shed light on important protein–lipid interactions and on the effect of lipid matrices on protein functionality.

## Supporting Information Available

Variation of the phase angle as a function of temperature for GMO, GMO/OA 10%, and GMO/OA 25% systems and second derivative ATR–FTIR spectra of Ins, GMO + Ins 4%, GMO/OA 10% + Ins 4%, and GMO/OA 25% + Ins 4% (PDF)
