# Supplementary material for: Cubic and Hexagonal Mesophases for Protein Encapsulation: Structural Effects of Insulin Confinement
Source: Langmuir. 2021 Aug 9;37(33):10166–76. doi: 10.1021/acs.langmuir.1c01587 (PMC8397388; doi:10.1021/acs.langmuir.1c01587)
Supplement: Supplementary file 1 — la1c01587_si_001.pdf [file la1c01587_si_001.pdf]

Supporting Information

Cubic and hexagonal mesophases for protein encapsulation: structural effects of insulin confinement

*Paola Astolfi,[1] Elisabetta Giorgini,[2] Diego Romano Perinelli,[3] Francesco Vita,[1] Fabrizio Corrado Adamo,[1] Serena Logrippo,[1] Marco Parlapiano,[1] Giulia Bonacucina[3], Stefania Pucciarelli,[4] Oriano Francescangeli,[1] Lisa Vaccari,[5] Michela Pisani[1]**

[1] Dipartimento SIMAU, Università Politecnica delle Marche, via Brecce Bianche, 60131 Ancona, Italy

[2] Dipartimento DISVA, Università Politecnica delle Marche, via Brecce Bianche, 60131 Ancona, Italy

[3] Scuola di Scienze del Farmaco e dei Prodotti della Salute, Università di Camerino, Via Gentile III da Varano, 62032 Camerino, Macerata, Italy

[4] Scuola di Bioscienze e Medicina Veterinaria, Università di Camerino, Via Gentile III da Varano, 62032 Camerino, Macerata, Italy

[5] Elettra - Sincrotrone Trieste S.C.p.A., S.S. 14 - km 163.5, 34149 Basovizza, Trieste, Italy

Corresponding author: m.pisani@univpm.it

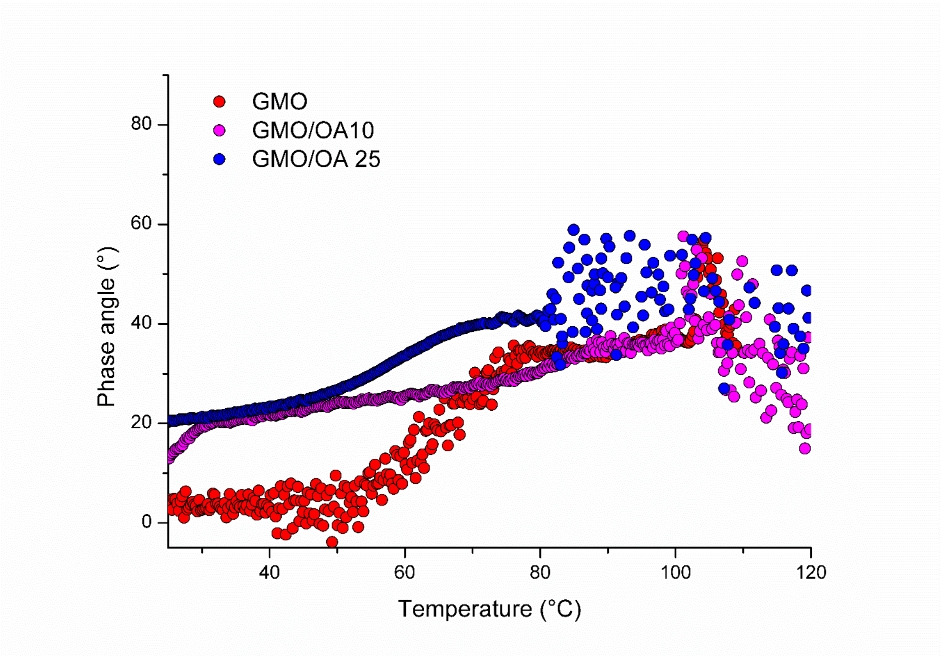

Fig S1 Variation of the phase angle as a function of temperature for GMO, GMO/OA10% and GMO/OA25% systems (temperature sweep test)

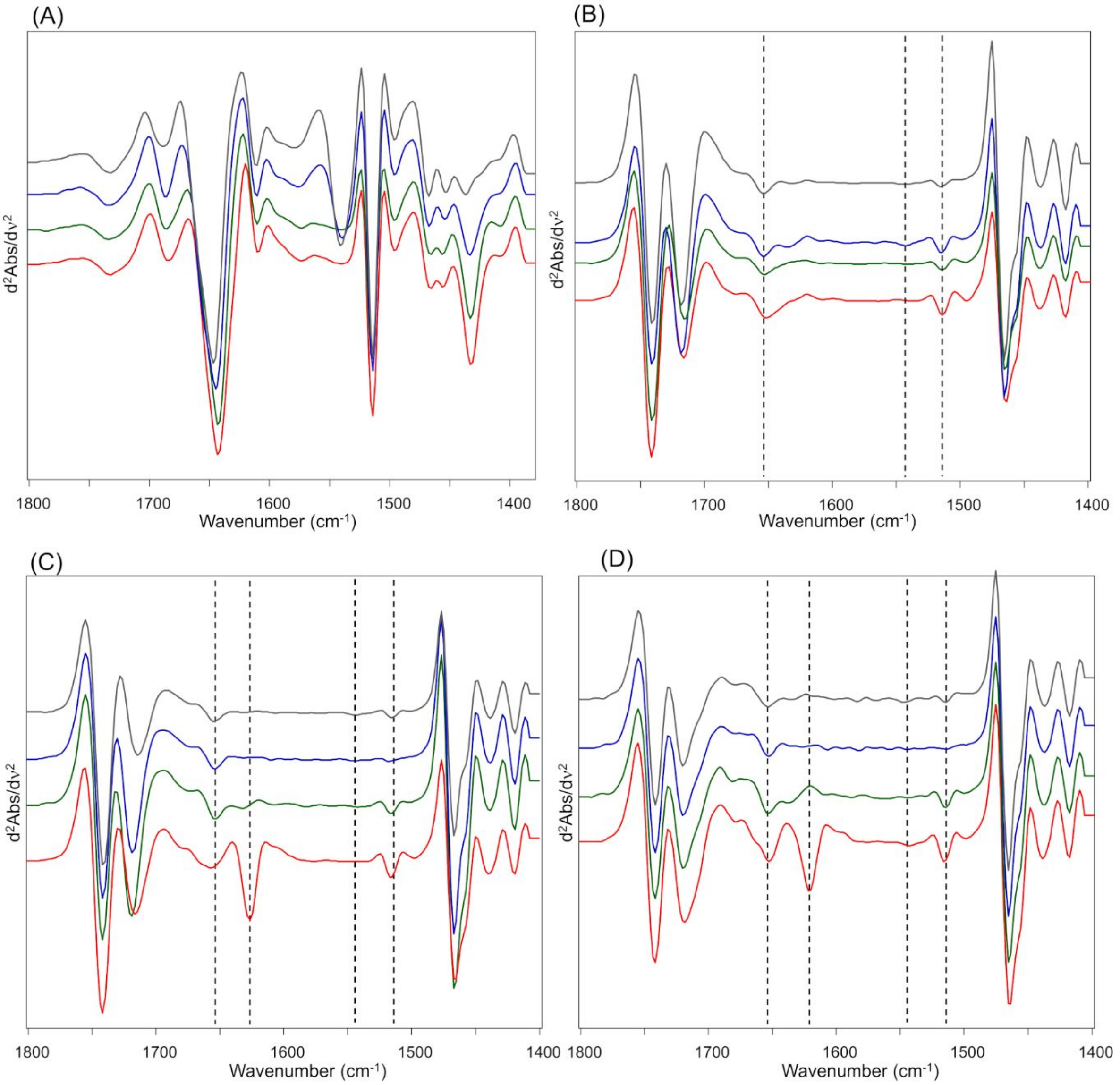

Fig S2 Second Derivative ATR-FTIR spectra of: (A) Ins; (B) GMO + Ins 4%; (C) GMO/OA10% + Ins 4%, and (D) GMO/OA25% + Ins 4%. Spectra are reported in the 1800-1400 cm^{-1} range and are

referred to spectra acquired at 25°C (grey), 45°C (blue), 55°C (green), and 70°C (red). DII IR spectra are shifted along y-axis for a better viewing. Dotted lines indicate the IR bands ascribable to insulin.
